# Integrin receptor-binding nanofibrous peptide hydrogel for combined mesenchymal stem cell therapy and nitric oxide delivery in renal ischemia/reperfusion injury

**DOI:** 10.1186/s13287-022-03045-1

**Published:** 2022-07-26

**Authors:** Haniyeh Najafi, Samira Sadat Abolmaali, Reza Heidari, Hadi Valizadeh, Ali Mohammad Tamaddon, Negar Azarpira

**Affiliations:** 1 Pharmaceutical Nanotechnology Department, Shiraz University of Medical Sciences, P. O. Box 71345-1583, Shiraz, Iran; 2 Center for Nanotechnology in Drug Delivery, Shiraz University of Medical Sciences, P. O. Box 71345-1583, Shiraz, Iran; 3 Pharmaceutical Sciences Research Center, Shiraz University of Medical Sciences, P. O. Box 71345-1583, Shiraz, Iran; 4 Drug Applied Research Center and Faculty of Pharmacy, Tabriz University of Medical Science, P. O. Box 51369-43738, Tabriz, Iran; 5 Transplant Research Center, Shiraz University of Medical Sciences, Mohammad Rasoul-Allah Research Tower, P. O. Box 7193711351, Shiraz, Iran

**Keywords:** Dipeptide hydrogel, RGD peptide, Mesenchymal stem cells, Nitric oxide donor, Renal ischemia/reperfusion injury

## Abstract

**Background:**

Mesenchymal-based therapy has been utilized as a practical approach in the treatment of renal ischemia/reperfusion (I/R) injury. However, low cell retention and survival in the ischemic site have remained challenging issues. To bridge this gap, the integrin receptor-binding RGD peptide-functionalized, s-nitroso-n-acetyl penicillamine (SNAP)-loaded hydrogel was used to transplant Wharton's jelly-mesenchymal stem cells (WJ-MSCs).

**Methods:**

Apart from physicochemical and rheological characterizations that confirmed entangled interlocking β-sheets with nanofibrous morphology, real-time RT-PCR, ROS production, serum biomarker concentrations, and histopathological alterations were explored in a mouse model to assess the therapeutic efficacy of formulations in the treatment of renal I/R injury.

**Results:**

The RGD-functionalized Fmoc-diphenylalanine (Fmoc-FF + Fmoc-RGD) hydrogel supported the spread and proliferation of WJ-MSCs *in vivo*. Notably, intralesional injection of nitric oxide donor combined with the embedded WJ-MSCs caused superior recovery of renal I/R injury compared to free WJ-MSCs alone in terms of histopathological scores and renal function indices. Compared to the I/R control group, oxidative stress and inducible nitric oxide synthase (iNOS) expression biomarkers showed a significant decline, whereas endothelial nitric oxide synthase (eNOS) and vascular endothelial growth factor (VEGF) expression exhibited a significant increment, indicating regeneration of the injured endothelial tissue.

**Conclusion:**

The findings confirmed that the hydrogels containing WJ-MSCs and nitric oxide donors can promote the regeneration of renal I/R injuries by increasing angiogenic factors and cell engraftment.

**Supplementary Information:**

The online version contains supplementary material available at 10.1186/s13287-022-03045-1.

## Introduction

Ischemia/reperfusion (I/R) injury is the leading cause of transplant failure, imposing a huge economic burden as well as declining the quality of patients’ lives [1]. Recently, mesenchymal-based therapy has attracted increasing attention as a promising strategy for I/R treatment due to its immunomodulatory, angiogenesis, anti-inflammatory, and homing properties [2]. However, its applications are limited due to the low rate of cell engraftment, poor delivery to target tissues, and low survival [3]. Because of these obstacles, developing methods for alleviating cell apoptosis may be of the utmost importance for mesenchymal-based therapy. The strategy to transplant stem cells with synthetic biomimetic scaffolds provides not only a scaffold for cell anchorage but also a supportive niche for cell engraftment [4, 5]. Over the last decades, self-assembling peptides (SAPs) have attracted a huge deal of attention due to their valuable features such as biocompatibility, excellent injectability, mechanical robustness, ability to load cells and active agents, tailoring functional behavior, and facile folding to form 3D biomimetic matrix [6–10]. Moreover, SAPs are ideal templates for stem cell therapy as they can mimic the extracellular environment. Recently, Huang et al*.* prepared a novel bioactive hydrogel, Nap-GFFYK-Thiol, through disulfide bonding as cleavable linkers for controlling the molecular self-assembly in a mouse ischemic hindlimb model. Their results revealed that human placenta-derived mesenchymal stem cells (hP-MSCs) transplanted with Nap-GFFYK-Thiol hydrogel could ameliorate limb ischemia by promoting hP-MSC survival and secretion of proangiogenic factors [11]. Similarly, a functional hydrogel was developed by Wang et al*.* through linking naphthalene (Nap) to a short peptide (Nap-DFDFG) and the insulin-like growth factor-1 (IGF-1). Their results indicated that co-transplantation of the β-IGF-1 hydrogel and hP-MSCs boosted angiogenesis in a murine acute kidney injury [7] model, leading to recovery of renal function [12].

Since combination therapy in ischemic diseases is presumed to be more effective than a single MSCs-based therapy, various agents including pharmaceuticals, bioactive molecules, and other cell types have been combined with MSCs to treat ischemic diseases [13–15]. Nitric oxide (NO) could regulate cell proliferation, differentiation, and migration, in addition to serving as an essential gaseous signaling molecule [16, 17]. NO is capable of promoting tissue regeneration by enhancing self-renewal ability and inhibiting the mal-differentiation of mesenchymal progenitors [18]. NO utilization in renal I/R injury treatment is, however, limited by its short half-life, severe toxicity, and low solubility [19]. These challenges can be overcome by a continuous and prolonged NO release. Zhang et al. designed NO-releasing chitosan hydrogel co-transplanted with hP-MSCs in the hindlimb ischemia model. Using the bioluminescence imaging technique, they revealed that controlled NO release can improve the grafting of hP-MSCs and ameliorate the functional recovery of ischemic hindlimbs [20]. Similarly, the therapeutic efficiency of MSCs in the murine myocardial infarction models can be enhanced by the NO-releasing naphthalene-modified short peptide hydrogel [21].

Recently, an injectable NO-releasing hydrogel was developed based on short aromatic dipeptides through physical crosslinking. Thanks to the localized and sustained release of NO, the Fmoc-diphenylalanine S-nitroso-N-acetyl penicillamine hydrogel (Fmoc-FF-SNAP) could better mediate the regeneration of renal I/R injuries and improve renal function compared to SNAP alone [22]. The present paper reports the development of a novel RGD-functionalized SNAP-loaded hydrogel (Fmoc-FF + Fmoc-RGD-SNAP) based on hierarchical nanofibrous self-assembly of peptide precursors for mesenchymal cell therapy in the acute renal ischemia mouse models. To this aim, Fmoc-FF was decorated with Fmoc-RGD, which had been synthesized through solid-phase synthesis to provide a subtle microenvironment for encapsulating WJ-MSCs [23, 24]. The hydrogel was tested in 3D culture, and the DiD-labeled cells embedded in the hydrogel were imaged after intralesional injection to track the cells in the kidney. The therapeutic efficacy of Fmoc-FF + Fmoc-RGD-SNAP-MSCs in the renal I/R injury model was explored relative to free MSCs and Fmoc-FF + Fmoc-RGD-SNAP by comparing kidney function biomarkers (e.g., blood urea nitrogen (BUN) and serum creatinine (SCr) levels, reactive oxygen species (ROS) production, histopathological changes, and expression of vascular endothelial growth factor (VEGF), endothelial nitric oxide synthase (eNOS), and inducible nitric oxide synthase (iNOS)) were compared with the control groups.

## Materials and methods

### Chemicals

Fmoc-phenylalanine and tert-butyl ester amino acid (NH_2_-phenylalanine-OtBu) were supplied by Chempep (Wellington, Florida, USA) and Aapptec (Louisville, Kentucky, USA). Tert-butyl methyl ether, piperidine, phosphoric acid, formalin, and chloroform (CHCl_3_) were supplied by Merck (Darmstadt, Germany). TRIzol Reagent kit and trifluoroacetic acid (TFA) were purchased from Life Technologies (Rockville, Md, USA) and Daejung (Busan, South Korea), respectively. Fmoc-RGD and Fmoc-RGE were prepared from Abcam (Cambridge, UK). S-Nitroso-N-Acetyl-D, L-Penicillamine (SNAP), and Prime Script RT Reagent Kit were supplied by Santa Cruz (Dallas, Texas, USA) and Takara (Tokyo, Japan). Alamar blue viability assay kit was purchased from G-biosciences (St Louis, MO, USA). DiD labeling solution and commercial kits for evaluating serum biomarkers were supplied by AAT Bioquest (Sunnyvale, California, USA) and Man (Tehran, Iran), respectively. Primary and secondary antibodies including anti-mouse iNOS, anti-mouse eNOS and anti-mouse VEGF antibody and Fmoc-RGD (Fmoc-RGE) were provided from Abcam (Cambridge, MA, USA). N-methyl morpholine (NMM), 3-(4,5-dimethylthiazol-2-yl)-2,5-diphenyltetrazolium bromide (MTT), fluorescein diacetate (FDA), propidium iodide (PI), isobutyl chloroformate, 2′,7′-Dichlorofluorescein diacetate (DCFH-DA), and Coomassie brilliant blue were supplied from Sigma-Aldrich (St Louis, MO, USA). All aqueous solutions were freshly prepared with deionized water (Direct Q UV3, Millipore, USA).

### Animals care

Male BALB/c mice (*n* = 45, weighing 20–25 g) were supplied from Animal Breeding Center, Shiraz University of Medical Sciences, Shiraz, Iran. They were kept at ambient temperature 24 ± 1 °C with a relative humidity of 50 ± 5. The mice had free access to a standard pellet chow (Behparvar®, Tehran, Iran) and tap water. The animals were kept according to the guidelines regarding the care and use of laboratory animals approved by the institutional ethics committee at Shiraz University of Medical Sciences, Shiraz, Iran (IR.SUMS.REC.1396.S982).

### Peptide hydrogel preparation and characterization

Fmoc-FF (Chempep, USA) was synthesized through tert-butyl ester reaction chemistry, as reported elsewhere [25]. Briefly, 315 µl isobutyl chloroformate was added to the Fmoc-phenylalanine-OH (472 mg) solution in CHCl_3_ under stirring. The mixture was then stirred overnight by adding 2.5 ml phenylalanine-OtBu (314 mg) to CHCl_3_. Upon dissolving Fmoc-FF in a CHCl_3_/TFA (1:1) mixture, the tert-butyl protecting groups were removed. The product was precipitated with cold tert-butyl methyl ether, centrifuged for 15 min at 3000 × g, and lyophilized (Christ alpha 1–2 LD, Germany).

To prepare the mixed peptide hydrogels, Fmoc-FF + Fmoc-RGD or Fmoc-FF + Fmoc-RGE, 20 mM Fmoc-FF (pH = 10) and 20 mM Fmoc-RGD (Fmoc-RGE) (pH = 3) were mixed in 15% and 30% volume ratios and vortexed until homogenization. The mixed solution was further neutralized to pH = 7 by dropwise addition of dilute HCl or NaOH. Field-emission scanning electron microscopy (FE-SEM) was carried out using a HITACHI S-4160 microscope (Japan) to investigate the nanoscale morphology of the mixed hydrogels.

### Oscillatory rheology

Dynamic frequency sweep experiments on an Anton Paar (MCR-302) rheometer equipped with cone-plate (24.983 mm/1.002 diameters) or cup-bob (16.661 mm–18.088 diameters) accessories were used to determine the mechanical properties of Fmoc-FF + 30%Fmoc-RGD hydrogel. The sample hydration was maintained by a solvent trap and an integrated temperature controller. An amplitude sweep was performed to ensure that the measurements were made in the linear viscoelastic zone, and the results showed no variation in the elastic (G′) and viscus (G″) moduli up to a strain of 1%. Frequency sweep test (0.1 and 100 rad.s^−1^) was performed to record variations in G′ and G″ moduli at pH of 8.5 (before gelation) and 7.4 (in situ gelation) at 4 °C and 37 °C. Moreover, the hydrogel was tested at various temperatures at pH levels of 8.5 and 7.4. The temperature was first raised from 4 to 90 °C at a rate of 5 °C.min^−1^, then maintained at 90 °C for 1.5 min. The temperature dropped from 90 to 4 °C in 1 min before remaining at 4 °C for another 8 min. All the experiments were carried out in triplicates.

### 3D-cell culture of WJ-MSCs

Before preparing peptide solutions, Fmoc-FF, Fmoc-RGD, and Fmoc-RGE were individually weighed and sterilized by UV light for 30 min. Fmoc-FF, Fmoc-FF + 15%&30%Fmoc-RGD, and Fmoc-FF + 15&30%Fmoc-RGE solutions (20 mM) were separately prepared, and pre-heated in an incubator at 37 °C. Tissue culture inserts were loaded up with an equal volume of solutions and WJ-MSC suspensions (10^5^ cells/well) and placed in a 12-well microplate (Orange Scientific, Belgium) to encapsulate the cells. The mixtures were gently shaken to ensure homogeneity; 2 ml of complete medium was added to the wells and incubated for 30 min. An additional 200 µl of complete medium was added to cover the peptide scaffolds. The medium surrounding the peptide scaffold was daily refreshed for 2 days.

### Live-Dead fluorescence microscopy

The FDA (green)/PI [26] double-staining assay was employed to monitor the possible death of the encapsulated cells in the 3D hydrogel scaffold using a fluorescent microscope [26]. The medium was withdrawn at specified times (24 and 72 h post-incubation), and the cell-gel constructs were rinsed with phosphate-buffered saline (PBS). The cell-encapsulated scaffolds were then stained with FDA/PI mix in PBS and observed under a fluorescent microscope (Olympus CKX53, Japan). All experiments were repeated at least three times.

### MTT cytotoxicity assay

MTT cytotoxicity assay was carried out to investigate the cytotoxicity of the peptide scaffolds [27]. WJ-MSCs were seeded in 96-well plates at a similar density of 15 × 10^3^ cells/well. After 24 h of incubation, the cells were treated with serum-free culture media containing various concentrations of Fmoc-FF, Fmoc-FF + 15%&30%Fmoc-RGD, and Fmoc-FF + 15&30%Fmoc-RGE solutions (0.001–0.5 µM) and incubated at 37 °C and 5% CO_2_ for another 24 h. The medium was then replaced with 100 µl MTT (0.5 mg/ml) in PBS (pH 7.4). After 3 h, the medium was aspirated, and the remaining formazan crystals were solubilized in DMSO (100 µl/per well). Optical absorbance was measured at *λ* = 570 nm (test) and corrected for the background absorbance at *λ* = 650 nm (reference) using a microplate reader. Cell viability (%) was calculated relative to control (untreated) cells.

### Alamar blue cell proliferation assay

The proliferation of encapsulated cells in the peptide scaffolds was determined by Alamar blue assay according to a previously published method [28]. A 3D culture was performed as described in Sect. 2.5. At specific times (4, 24, and 72 h post-incubation), the culture medium was aspirated, the cell-gel constructs were washed with sterile PBS buffer (pH = 7.4) and incubated with 10% (*v*/*v*) fresh Alamar blue in the complete medium for further 2 h. The fluorescence intensity was read by the fluorescence plate reader (Infinite 200, Tecan, Austria) at the respective excitation and emission wavelengths of *λ*_max_ = 545 and 590 nm.

### Animal model and study groups

To develop the renal I/R model in mice subjects, the right kidney was removed, then, the left renal pedicle was clamped for 45 min followed by reperfusion [22]. Following renal ischemia induction, male mice were randomly divided into 9 groups (*n* = 5) and received an intra-renal parenchymal injection at three different sites immediately after reperfusion. The models received the following treatments: (1) normal mice control (Norm); (2) sham control (I/R group which received normal saline, M); (3) I/R + Fmoc-FF/30%Fmoc-RGD; 4) I/R + 15 µM SNAP (SM); (5) I/R + 15 µM SNAP + Fmoc-FF/30%Fmoc-RGD (SHM); (6) I/R + 1 × 10^6^ WJ-MSCs (CM); (7) I/R + 1 × 10^6^ WJ-MSCs + Fmoc-FF/30%Fmoc-RGD (CHM); (8) I/R + 1 × 10^6^ WJ-MSCs + 15 µM SNAP (SCM); and (9) I/R + 1 × 10^6^ WJ-MSCs + 15 µM SNAP + Fmoc-FF/30%Fmoc-RGD (SCHM). At the end of the experiment (Day 7), the animals were anesthetized (ketamine/xylazine; 100/10 mg/kg, i.p.) to collect their blood and kidney samples.

#### Tracking WJ-MSCs in renal I/R model

MSCs were labeled with DiD, a lipophilic cyanine near-infrared fluorochrome, to track their translocation in the kidney. Briefly, 1 × 10^6^ WJ-MSCs were incubated with 5 μM DiD dye for 20 min. The cells were then rinsed twice with PBS before being injected into renal I/R mice with and without Fmoc-FF + 30%Fmoc-RGD peptide hydrogel. Mice were imaged on Day 7 using an *in vivo* imaging system equipped with Cy5.5 filter set (excitation filter: 630 nm, emission filter: 700 nm, exposure time: 10 s, F-stop: 2.5, FOV: 84.73 mm) [29].

#### Serum biochemistry

Blood was sampled from the abdominal aorta on Day 7, it was then transferred to standard tubes (Improvacuter®; gel and clot activator-coated tubes; Guangzhou, China) and centrifuged (3000 × g, 10 min, 4 °C) to prepare the serum. Mindray BS-200® autoanalyzer and commercial kits were employed to measure SCr and BUN levels [30].

#### ROS measurement

Kidney samples (100 mg) were homogenized in ice-cooled KCl buffer (40 mM, pH = 7.4) (1:10 w/v). The tissue homogenates (100 µl) were then combined with 1 ml Tris–HCl buffer (40 mM, pH = 7.4) and 5 µl 2′, 7′-dichlorofluorescein diacetate (2 mM). Subsequently, the mixture underwent 30 min of incubation in darkness at 37 °C (Gyromax™ incubator shaker). Ultimately, a FLUOstar Omega® multifunctional microplate reader (*λ*_excitation_ = 485 nm and *λ*_emission_ = 525 nm) was utilized to assess the fluorescence intensity of the samples [31].

#### Kidney histopathology

Histopathological assessments were carried out by fixing the kidney tissue samples in a formalin buffered solution (0.4% sodium phosphate monobasic, NaH_2_PO_4_, 0.64% sodium phosphate dibasic, Na_2_HPO_4_, and 10% formaldehyde in deionized water, pH = 7.4). The paraffin-embedded Sects. (5 µm) of the tissue were stained with hematoxylin and eosin (H&E) [32]. Renal histopathological alterations were scored according to a previously described procedure [22]. A pathologist blindly analyzed the samples using a light microscope (Olympus®, Japan) [33].

#### RNA isolation and real-time PCR

Total RNA was extracted from 50 to 100 mg of the kidney samples using TRIzol Reagent according to the manufacturer’s instructions to assess the expression of eNOS (NM_008713.4), iNOS (NM_010927.4), VEGF (NM_001025250.3), and glyceraldehyde-3-phosphate dehydrogenase (GAPDH) (NM_017008.4) genes. The primer sequences were as follows: eNOS: 5′-AACCATTCTGTATGGCTCTGAGAC-3′, 5′-CTCTAGGGACACCACATCATACTC-3′; iNOS: 5´-ATGTGCTGCCTCTGGTCTT-3′, 5′-CCTGGAACCACTCGTACTTG-3′; VEGF: 5′-GTCCTCTCCTTACCCCACCT-3′, 5′-CACACACAGCCAAGTCTCCT-3′; GAPDH: 5′-AACGACCCCTTCATTGAC-3′, 5′-AGGGAAATCGTGCGTGAC-3′. NanoDrop™ was used to analyze the RNA quality by measuring the optical density (260/280). The total RNA was then reverse transcribed into cDNA using the Prime Script RT Reagent Kit, as described by the manufacturer. Primers were generated by Allele ID 7 and Oligo 7 software (Premier Biosoft International, Palo Alto, USA). For data normalization, the mice GAPDH (housekeeping) gene was used as a reference. The expression levels of eNOS, iNOS, and VEGF were determined by the Livak (2^−ΔΔCT^) method. Melt curves were also analyzed to confirm the specificity of the reaction.

#### Immunohistochemistry

Primary anti-mouse eNOS, anti-mouse VEGF, and anti-mouse iNOS (1:100) antibodies were used to detect eNOS, VEGF, and iNOS proteins in kidney samples. An appropriate primary antibody was added to the blocking buffer followed by overnight incubation at 4 °C. After washing and room temperature incubation with secondary antibody for 30 min, the sections were washed and incubated with diaminobenzidine (DAB) as a chromogen for 5 min. The sections were mounted on slides after counterstaining with hematoxylin. For each image, three fields of consistent staining were chosen. The protein expressions for at least four images were determined using Image J software based on a threshold value [33].

#### Statistics

The obtained results were recorded as mean ± standard deviation (SD) and analyzed by Prism Software ver. 6.0 (GraphPad, USA). Image J (https://imagej.nih.gov/ij/) was also employed for calculating inter-fiber spacing, nanofiber thickness, and protein expression (%) for IHC. Statistical significance of the results was explored by one-way ANOVA using Dunnett’s multiple comparison tests and Kruskal–Wallis through Dunn’s multiple comparisons as post hoc tests. Statistical significance was taken as *P* < 0.05.

## Results

### Preparation and characterization of hydrogels

**Fig. 1:**
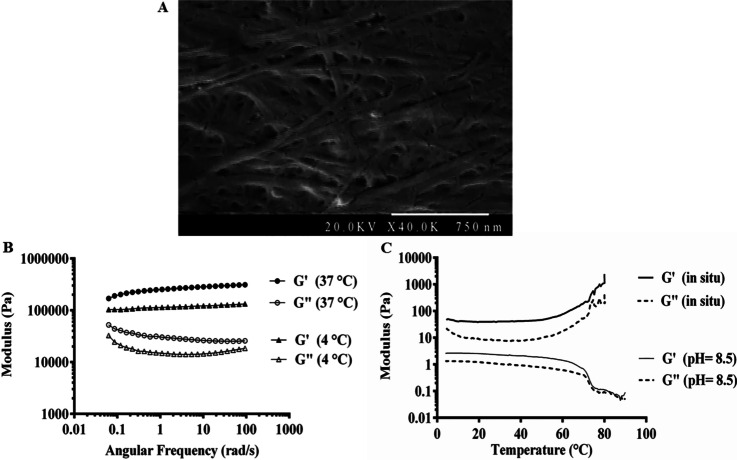
**A** FE-SEM image of self-assembled Fmoc-FF + 30%Fmoc-RGD hydrogel (thickness = 30 ± 2 nm). **B** Dynamic frequency sweep at 4 °C and 37 °C. **C** Dynamic temperature sweep from 4 to 90 °C

Fmoc-FF hydrogel was synthesized and characterized as described before [22]. The next step involved the FE-SEM imaging of Fmoc-FF + 30%Fmoc-RGD to confirm the formation of nanofibrous networks with fibril thickness of 30 ± 2 nm (Fig. 1A).

A dynamic frequency sweep was first performed at 4 °C and 37 °C to investigate the rheological properties of the resulting hydrogels. As shown in Fig. 1B, G′ values at 4 °C were found to be 1.5–3 times higher than the temperature of 37 °C, indicating the stiffened Fmoc-FF + 30% Fmoc-RGD hydrogel upon heating. To further investigate the pH-temperature interplay on the mechanical moduli, a dynamic temperature sweep was obtained at pH of 8.5 and 7.4 (Fig. 1C). At pH of 8.5, the G′ values appeared to be approximately twice as high as the G″ values until a crossover of the moduli occurred around 70 °C (*T*_gel_). At pH of 7.4, several times higher values of G′ were observed compared to G″, suggesting the gelation at temperatures lower than the investigated range. Therefore, *T*_gel_ varies from below 4 °C to 70 °C depending on the pH of the medium.

### 3D culture and live/dead assay

**Fig. 2:**
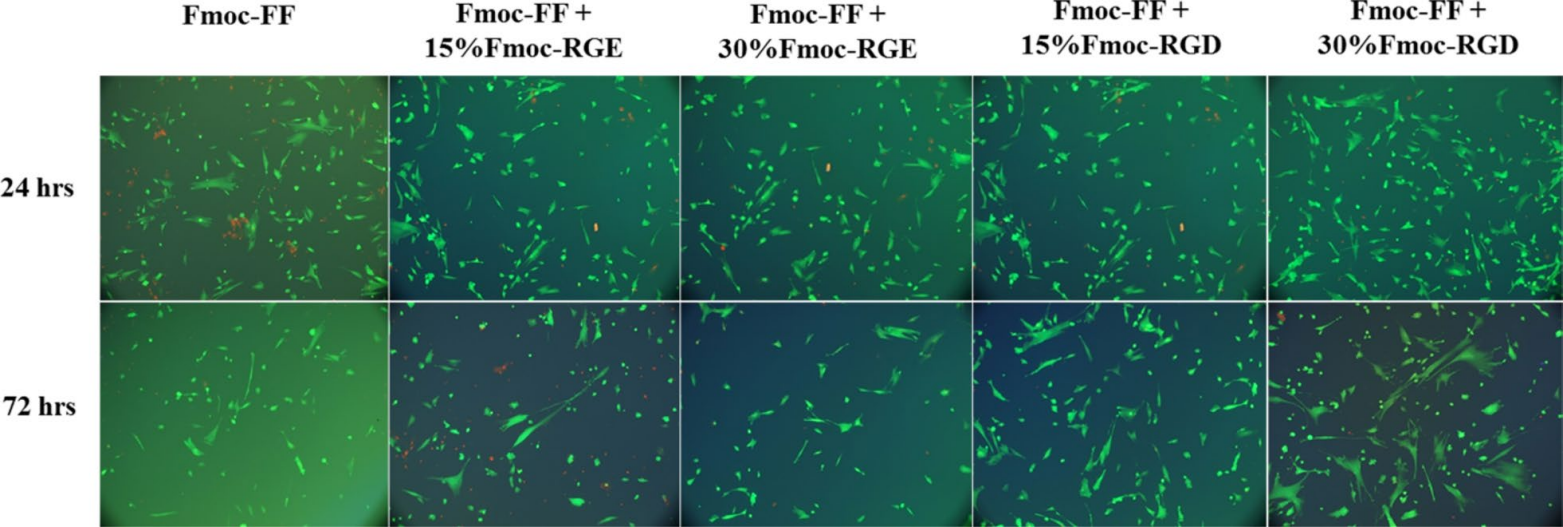
Fluorescence microscopy images of the 3-dimensional culture of WJ-MSCs cells incorporated in the Fmoc-FF, Fmoc-FF + Fmoc-RGD (15% and 30%), and Fmoc-FF + Fmoc-RGE (15% and 30%) scaffolds. Green live cells showed normal morphology, while red ones showed dead cells

The effect of Fmoc-RGD as a cell–matrix adhesion molecule was investigated by preparing Fmoc-FF hydrogels containing WJ-MSCs with various contents of Fmoc-RGD or the control sequence (Fmoc-RGE). WJ-MSCs were isolated, expanded, and characterized according to our previous report [25]. For cell encapsulation, a mixture containing an equal volume of Fmoc-dipeptide solution with cell suspensions was prepared in serum-free DMEM. As shown in Fig. 2, the effect of combining Fmoc-RGD with Fmoc-FF hydrogels was concentration-dependent, so that Fmoc-FF + 30%Fmoc-RGD hydrogel greatly enhanced the WJ-MSC spread compared with Fmoc-FF + 15%Fmoc-RGD or controls (Fmoc-FF without Fmoc-RGD). When exposed to 15% Fmoc-RGD in the scaffold, the WJ-MSCs mostly remained round or elliptical; however, upon exposure to 30% Fmoc-RGD, the cells formed a 3D network. Importantly, in Fmoc-FF + Fmoc-RGE (15% and 30%), neither cell proliferation nor 3D network development was observed.

### MTT and Alamar blue assays

**Fig. 3:**
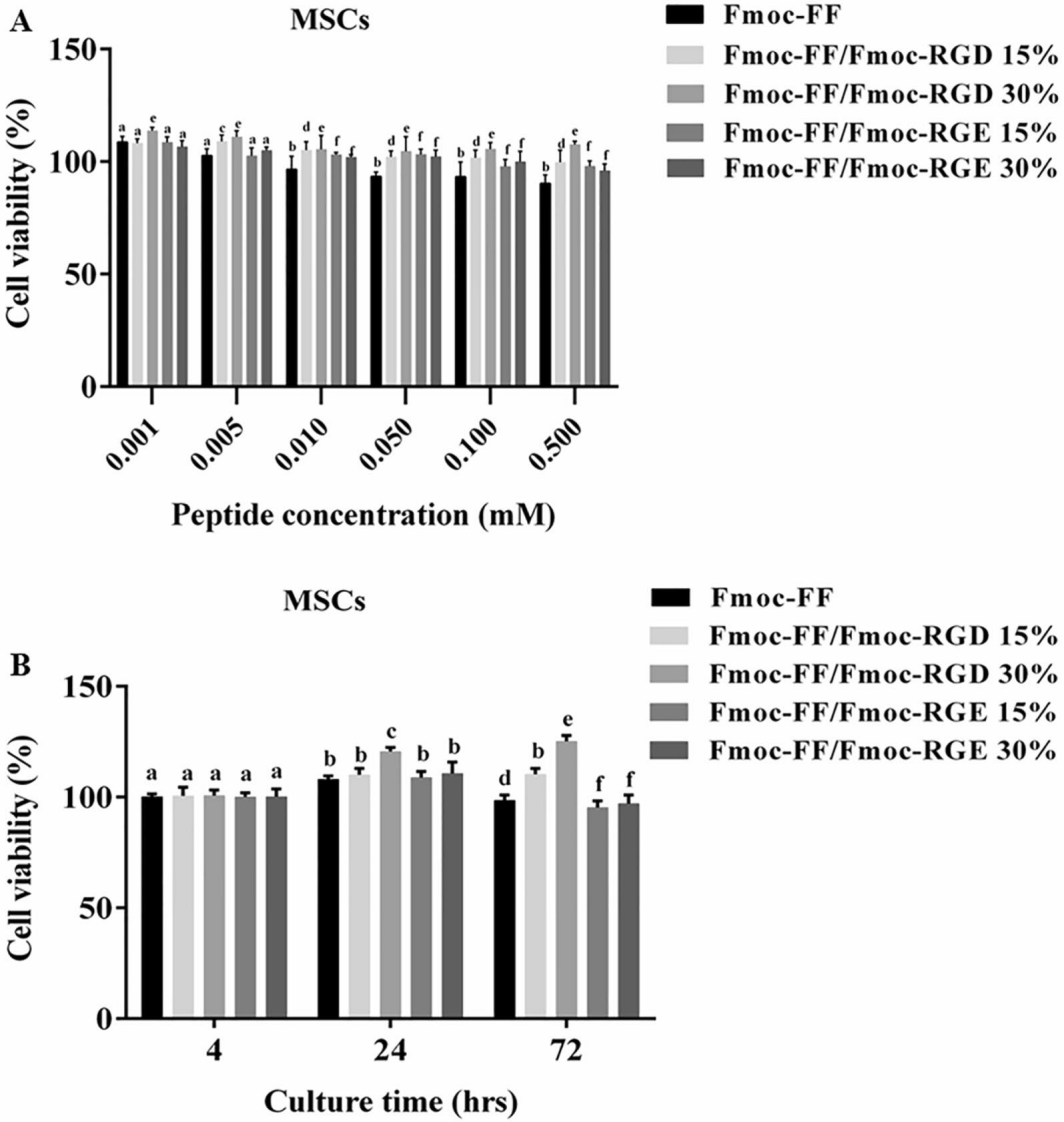
WJ-MSC viability in various concentrations of Fmoc-FF, either in combination with Fmoc-RGD, Fmoc-RGE, or alone (**A**) and after encapsulation in the 3D hydrogel scaffold for different incubation times (**B**). a, b, c, d, e, and f: inter-group comparisons according to the Tukey follow-up test; groups with the same superscripts were not significantly different at *α* = 0.05 (*P* > 0.05); however, dissimilar letters indicate a significant difference (*P* < 0.05)

The effect of the hydrogel constructs on cell viability and proliferation was explored by MTT and Alamar blue assays, respectively. Based on the MTT assay results in Fig. 3A, Fmoc-FF + 30%Fmoc-RGD nanofibers showed lower cytotoxicity at different concentrations when compared to Fmoc-FF (without RGD) or Fmoc-FF + 30%Fmoc-RGE. In another experiment, the proliferation of the WJ-MSCs was determined in the peptide hydrogel scaffolds through Alamar blue assay as depicted in Fig. 3B. Fmoc-RGD exhibited a concentration-dependent effect on WJ-MSC growth. Cell spreading and proliferation were significantly increased (by 20% after 3 days) in Fmoc-FF + 30%Fmoc-RGD compared to Fmoc-FF without Fmoc-RGD. Despite the modest increase in cell proliferation in Fmoc-FF + 15%Fmoc-RGD, the effects were not significant. No appreciable cell growth was found in combinations of Fmoc-FF and Fmoc-RGE (15% or 30%) (Fig. 3B).

### WJ-MSCs tracking

MSCs were DiD-labeled and injected into the parenchyma of the renal I/R mice. On Day 7, MSC homing was observed in the kidney organ, both alone and combined with the Fmoc-FF + 30% Fmoc-RGD. However, MSCs were more accumulated in the renal I/R of the CHM group than in the CM group. Furthermore, co-transplantation of MSCs with the peptide hydrogel declined the cell loss while enhancing cell proliferation compared to MSCs alone (Additional file 1).

### Biochemical markers and ROS measurement

**Fig. 4:**
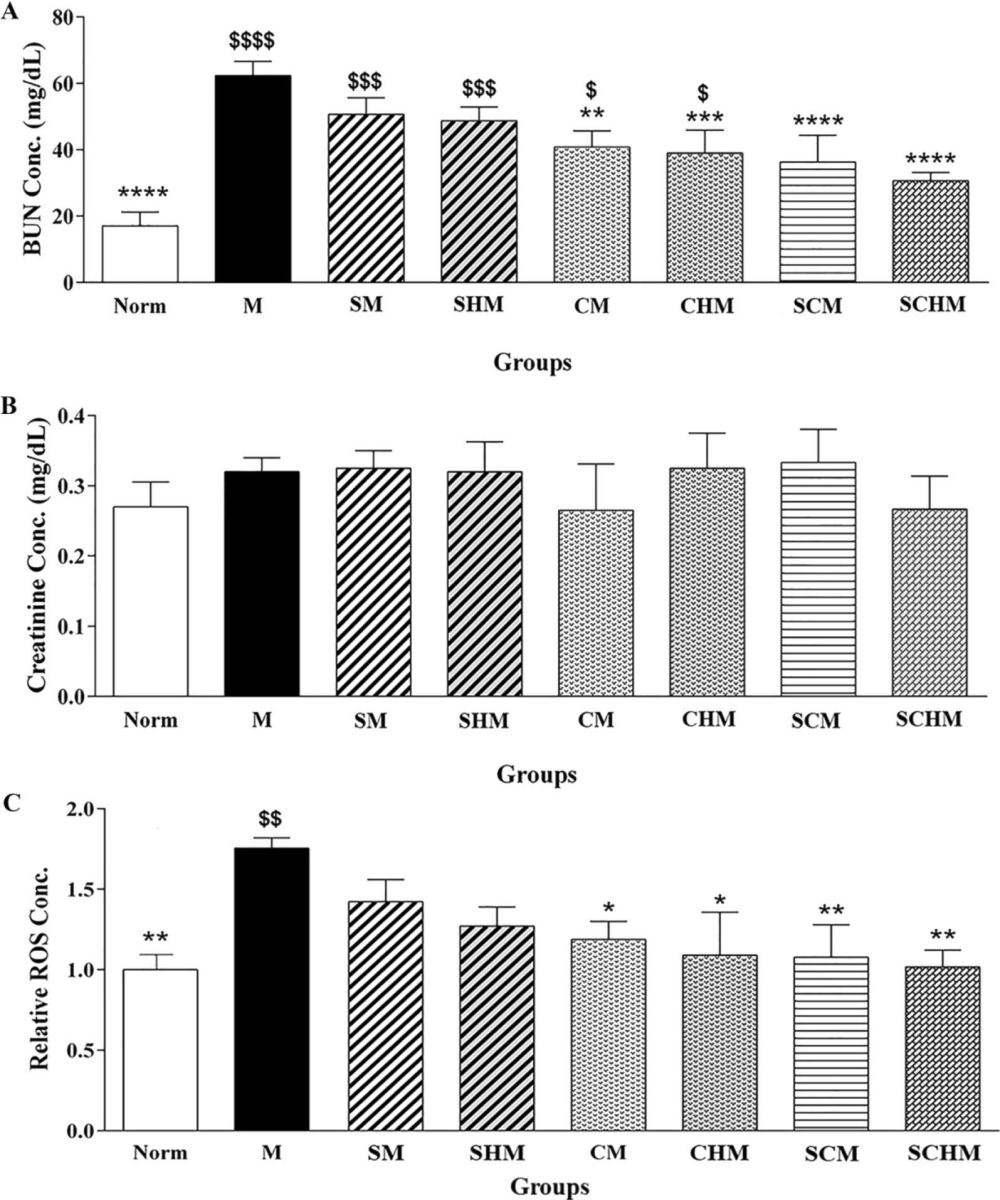
Serum biochemical and oxidative stress markers including BUN (**A**), serum creatinine (**B**), and ROS (**C**) in the renal ischemia–reperfusion (I/R) mice. The chart shows ROS formation based on DCF fluorescent intensity (**C**). Data are expressed as mean ± SD (*n* = 5). *P < 0.05 versus the control group (M),**P < 0.01 versus the control group (M), ***P < 0.001 versus the control group (M), ****P < 0.0001 versus the control group (M), $*P* < 0.05 versus the control group (Norm), $$*P* < 0.01 versus the control group (Norm), $$$P < 0.001 versus the control group (Norm), $$$$*P* < 0.0001 versus the control group (Norm). ROS: Reactive oxygen species. Control (normal mice, Norm), Control (Ischemia/reperfusion group which received normal saline, M), I/R + 15 µM SNAP (SM), I/R + 15 µM Fmoc-FF + 30%Fmoc-RGD-SNAP (SHM), I/R + 1 × 106 WJ-MSCs (CM), I/R + 1 × 106 WJ-MSCs + 15 µM Fmoc-FF + 30%Fmoc-RGD (CHM), I/R + 1 × 106 WJ-MSCs + 15 µM SNAP (SCM), I/R + 1 × 106 WJ-MSCs + 15 µM Fmoc-FF + 30%Fmoc-RGD-SNAP (SCHM)

BUN and SCr levels of all groups were compared on the 7th day. The mentioned markers showed a significant increment in the renal I/R mice (M) compared to the Norm group (Fig. 4). Moreover, BUN levels were significantly lower in the CM (*P* < 0.01) and CHM (*P* < 0.001) groups as compared with the M group (Fig. 4A). Importantly, co-transplantation of MSCs with SNAP (SCM, *P* < 0.0001) or Fmoc-FF + 30%Fmoc-RGD-SNAP (SCHM, *P* < 0.0001) reduced the BUN level more significantly than the M group (Fig. 4A). Despite a decline in SCr levels in the CM and SCHM, the difference was not statistically significant (Fig. 4B). Biomarker of ROS was also assessed in the kidney of normal and I/R mice (Fig. 4C). Significant ROS formation was detected in the M group. Furthermore, ROS level showed a dramatic decrease in the CM (*P* < 0.05) and CHM (*P* < 0.05) groups compared with the M group. Interestingly, when MSCs were co-transplanted with SNAP, especially in combination with Fmoc-FF + 30%Fmoc-RGD, the ROS level reduced more considerably as compared with the M group (*P* < 0.01) (Fig. 4C).

### Histopathology

**Fig. 5:**
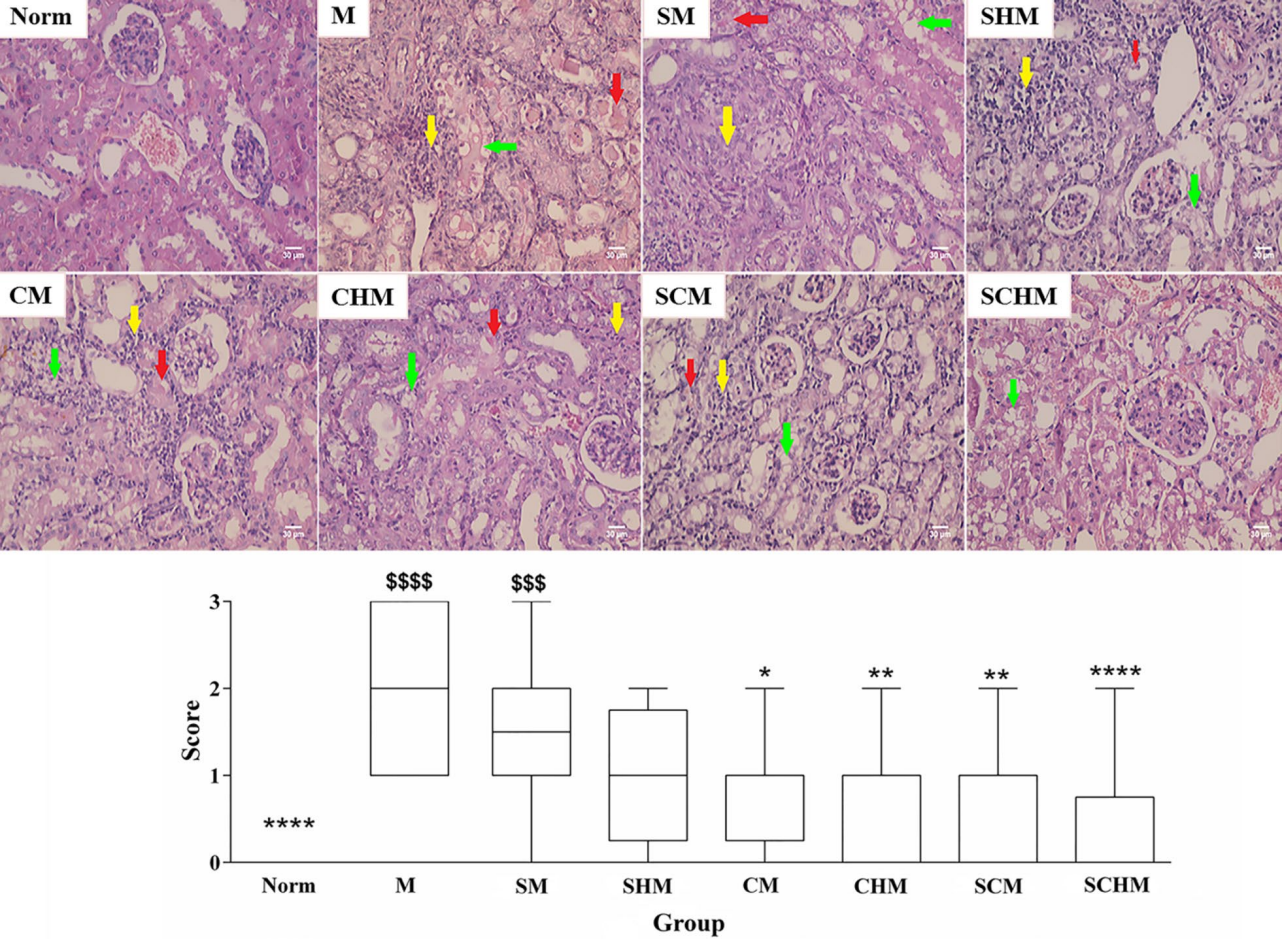
Histopathological alterations of kidney tissue in the renal ischemia–reperfusion (I/R) versus normal mice; up) H&E staining with × 400 magnification; down) Data for overall score of tissue histopathological changes including inflammation (yellow arrows), tubular cast (red arrows), and vacuolization (green arrows) were presented as mean ± SD (*n* = 5). *P < 0.05 versus the control group (M), **P < 0.01 versus the control group (M), ****P < 0.0001 versus the control group (M), $$$*P* < 0.001 versus the control group (Norm), $$$$*P* < 0.0001 versus the control group (Norm). Control (normal mice, Norm), Control (Ischemia/reperfusion group which received normal saline, M), I/R + 15 µM SNAP (SM), I/R + 15 µM Fmoc-FF + 30%Fmoc-RGD-SNAP (SHM), I/R + 1 × 106 WJ-MSCs (CM), I/R + 1 × 106 WJ-MSCs + 15 µM Fmoc-FF + 30%Fmoc-RGD (CHM), I/R + 1 × 106 WJ-MSCs + 15 µM SNAP (SCM), I/R + 1 × 106 WJ-MSCs + 15 µM Fmoc-FF + 30%Fmoc-RGD-SNAP (SCHM)

Inflammation, tubular cast, and vacuolization were among the histopathological alterations in the renal I/R mice (Fig. 5). Compared to the Norm group, the mentioned injuries showed higher scores in renal I/R mice. The renal I/R mice receiving WJ-MSCs exhibited markedly reduced glomerular and tubular damages compared to the M group (*P* < 0.05). Importantly, when SNAP and the peptide hydrogel were combined with WJ-MSCs, renal injuries were considerably decreased compared to the M group (*P* < 0.01). No evidence of kidney tissue damage was detected in SCHM mice as compared with the Norm group. Despite a decrement in the tubular and endothelial damages and inflammation in the other treated groups, their difference was not significant (Fig. 5). Specifically, when the peptide hydrogel or MSCs were combined with SNAP (SM), the histopathological scores significantly improved compared to the Norm and M groups.

### eNOS, iNOS, and VEGF gene expression

**Fig. 6:**
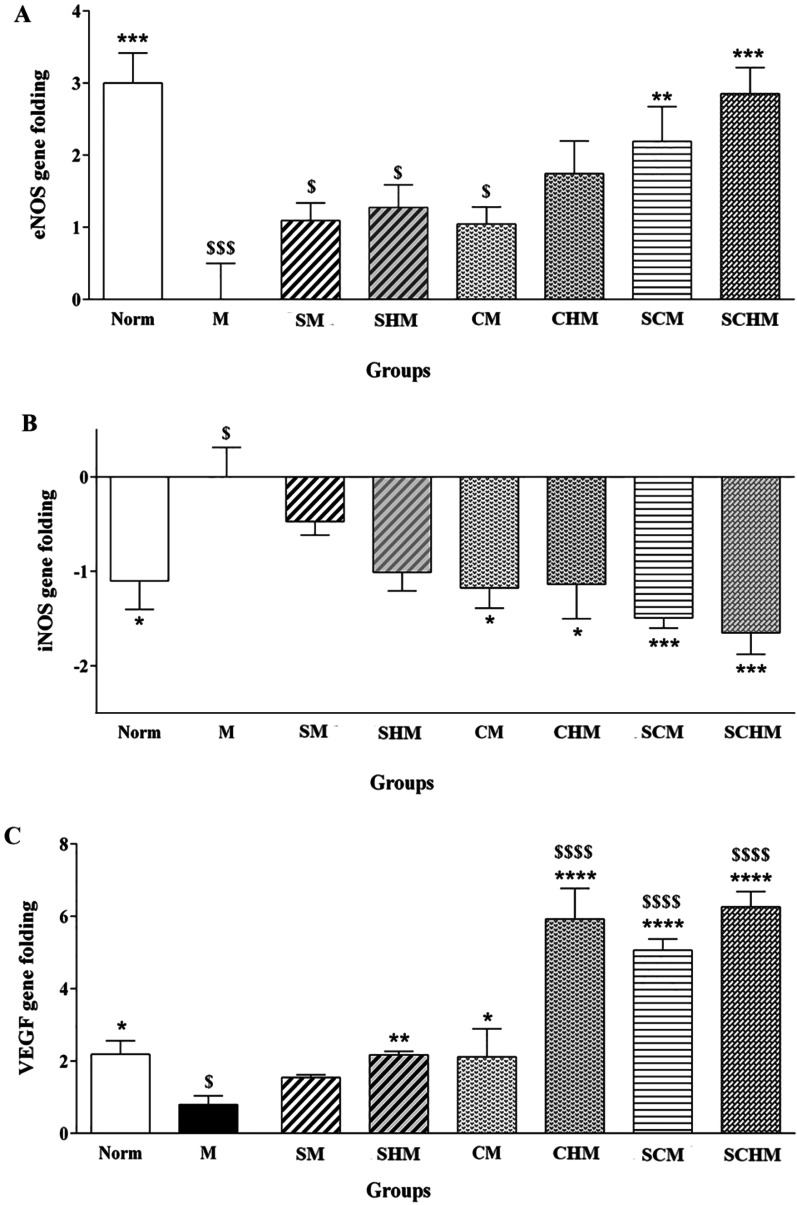
Gene expression analysis of eNOS (**A**), iNOS (**B**), and VEGF (**C**) in renal ischemia/reperfusion (I/R) mice. Data were expressed as mean ± SD (*n* = 5) *P < 0.05 versus the control group (M), **P < 0.01 versus the control group (M), ***P < 0.001 versus the control group (M), ****P < 0.0001 versus the control group (M), $*P* < 0.05 versus the control group (Norm), $$$*P* < 0.001 versus the control group (Norm), $$$$*P* < 0.0001 versus the control group (Norm). Control (normal mice, Norm), Control (Ischemia/reperfusion group which received normal saline, M), I/R + 15 µM SNAP (SM), I/R + 15 µM Fmoc-FF + 30%Fmoc-RGD-SNAP (SHM), I/R + 1 × 106 WJ-MSCs (CM), I/R + 1 × 106 WJ-MSCs + 15 µM Fmoc-FF + 30%Fmoc-RGD (CHM), I/R + 1 × 106 WJ-MSCs + 15 µM SNAP (SCM), I/R + 1 × 106 WJ-MSCs + 15 µM Fmoc-FF + 30%Fmoc-RGD-SNAP (SCHM)

The iNOS, eNOS, and VEGF expression profiles were significantly different in the M group compared to the Norm group (Fig. 6). The results showed that VEGF mRNA was up-regulated, and iNOS mRNA was down-regulated in almost all the treated groups compared to the I/R control group (Fig. 6B and C). Although iNOS gene expression in SNAP-treated groups decreased, the effect was not statistically significant (Fig. 6B). eNOS gene expression, on the other hand, was increased in all treated groups, however, this enhancement was only significant in SCM (*P* < 0.01) and SCHM (*P* < 0.001) compared to the M group (Fig. 6A). Overall, the enhancement of the eNOS and VEGF genes and the decrease in the iNOS gene were more statistically significant in SCHM mice (*P* < 0.001) compared to the M group. Interestingly, VEGF expression was significantly higher in CHM, SCM, and SCHM mice compared to the Norm subjects (*P* < 0.0001) (Fig. 6C), highlighting the synergic effect of WJ-MSCs and the peptide hydrogel on the induction of VEGF expression.

### eNOS, iNOS, and VEGF immunohistochemistry

**Fig. 7:**
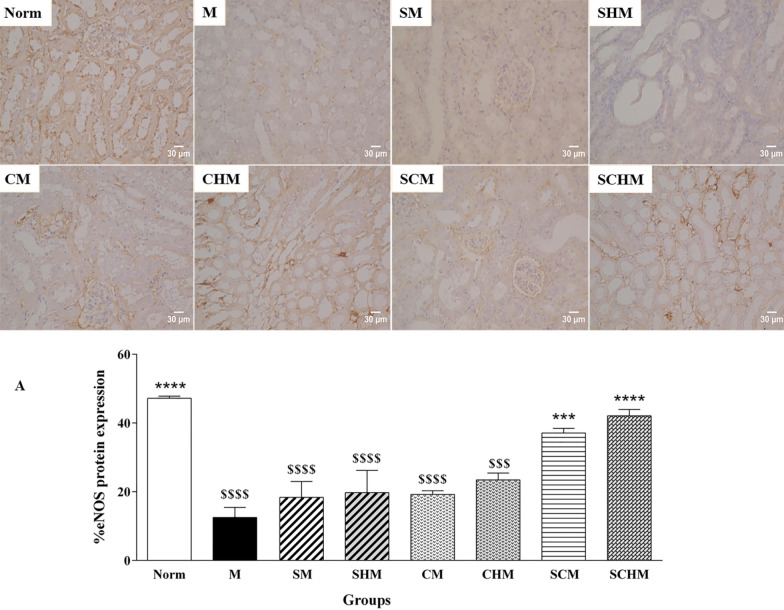

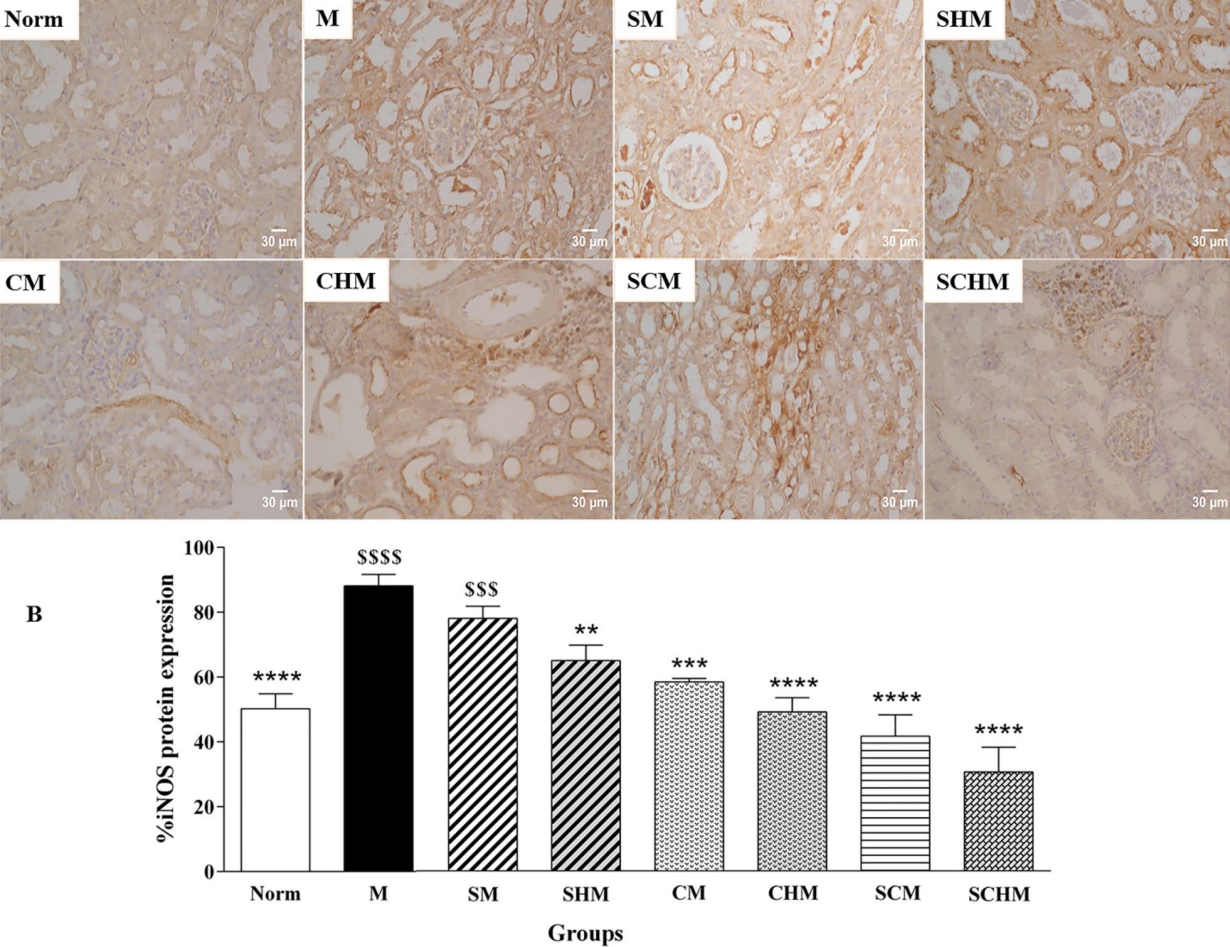

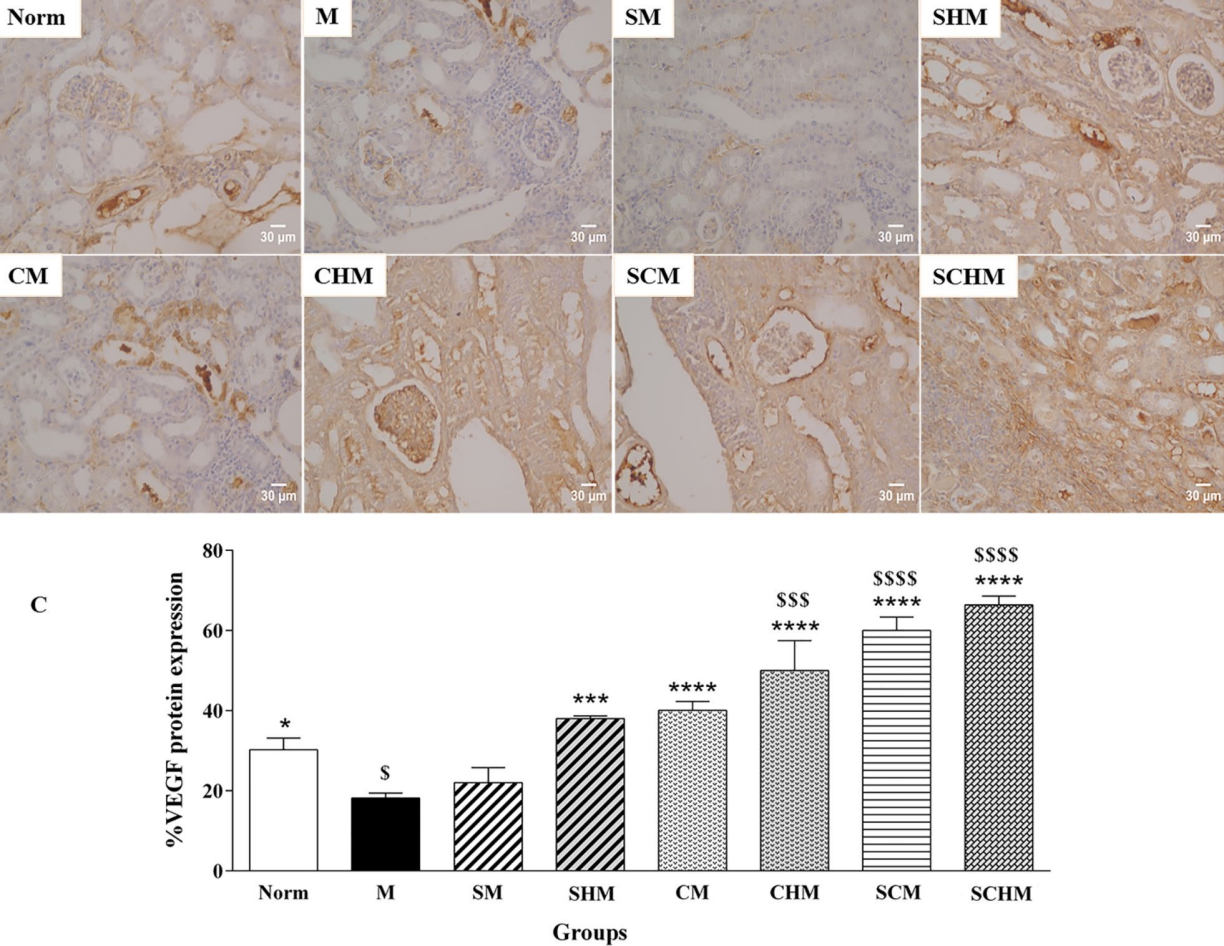
Protein expression of eNOS (**A**), iNOS (**B**), and VEGF (**C**) by immunocytochemistry in renal ischemia/reperfusion (I/R) mice. The charts show the protein expressions based on the intensity of the brown stained area in different groups with × 400 magnifications. Results were analyzed by Image J software. Data were represented as mean ± SD (n = 5). *P < 0.05 versus the control group (M), **P < 0.01 versus the control group (M), ***P < 0.001 versus the control group (M), ****P < 0.0001 versus the control group (M), $*P* < 0.05 versus the control group (Norm), $$$*P* < 0.001 versus the control group (Norm), $$$$*P* < 0.0001 versus the control group (Norm). Control (normal mice, Norm), Control (Ischemia/reperfusion group which received normal saline, M), I/R + 15 µM SNAP (SM), I/R + 15 µM SNAP-loaded Fmoc-FF + 30%Fmoc-RGD (SHM), I/R + 1 × 106 WJ-MSCs (CM), I/R + 1 × 106 WJ-MSCs + 15 µM Fmoc-FF + 30%Fmoc-RGD (CHM), I/R + 1 × 106 WJ-MSCs + 15 µM SNAP (SCM), I/R + 1 × 106 WJ-MSCs + 15 µM Fmoc-FF + 30%Fmoc-RGD-SNAP (SCHM)

Immunocytochemistry was performed to verify the combined effect of SNAP and MSCs on eNOS, iNOS, and VEGF protein expression on Day 7 (Fig. 7). In the renal I/R mice, a marked decline was detected in eNOS and VEGF expression while iNOS protein expression showed an increment compared to the Norm group. The eNOS protein increased in all treated groups, while this enhancement was only significant in SCM (P < 0.001) and SCHM (*P* < 0.0001) compared to the M group (Fig. 7A). Furthermore, the iNOS and VEGF levels exhibited a significant alteration in the groups treated with SNAP and MSCs (Fig. 7B and C). Interestingly, the expression of VEGF protein was significantly higher in the CHM, SCM, and SCHM groups compared to the M group (*P* < 0.0001) (Fig. 7C), suggesting that combining MSCs with Fmoc-FF + 30%Fmoc-RGD-SNAP resulted in enhanced effects.

## Discussion

I/R injury is a major problem in organ transplantation that can result in tissue dysfunction. Upon blood flow interruption, the kidney experiences hypoxia, elevated oxidative stress, and microvascular dysfunction. Additionally, subsequent reperfusion recruits and activates both the innate and adaptive immune systems, giving rise to progressive tissue damage [34, 35]. Despite the development of several methods to improve the I/R injury pathway, no optimal way has been yet introduced to resolve the negative effects of I/R injury on renal tissue. Mesenchymal cell-based therapy can improve renal function in I/R injury, but the extent of benefit of this approach is limited due to the poor retention and survival rate of transplanted stem cells [36, 37]. For example, more than 70% of administered stem cells were lost to the vasculature or squeezed back out of the injection site just two days post-intramyocardial injection [38]. A strategy involving combined biomaterial scaffolds with MSC therapy has been proposed to address this issue. This approach is appealing for controlled drug delivery and MSC homing after transplantation, as well as increased MSC retention and survival in an ischemic host microenvironment. Different injectable biomaterials are commonly used for stem cell therapy in tissue engineering, among which, alginate [39], chitosan [40], and cyclodextrin /MPEG–polycaprolactone–MPEG can be mentioned [41]. A novel Fmoc-FF-SNAP hydrogel was recently developed for sustained NO release at high SNAP concentrations (60 µM), addressing the influence of this system in a renal I/R model compared to free SNAP [22]. In the present study, the potential role of WJ-MSCs transplanted with new SNAP-loaded Fmoc-FF + Fmoc-RGD hydrogel in attenuating renal I/R injury was investigated and compared in a mouse model.

Before MSC incorporation, the morphological and rheological properties of Fmoc-FF + Fmoc-RGD hydrogels were explored. FE-SEM images verified the nanofibrous networks with fibril thickness of 30 ± 2 nm and proper porosity as a consequence of high hydrophobicity and agglomeration of Fmoc-FF + 30%Fmoc-RGD precursors (Fig. 1A). Based on our previous report, both Fmoc-FF and Fmoc-FF + 30%Fmoc-RGD contained similar flat ribbons with fibril thicknesses of ~ 30 nm [22]. Notably, the mechanical features (i.e., stiffness, recovery rate, and injectability) of hydrogel scaffolds can play a key role in regulating the interactions between cells and extracellular matrix (ECM) and directing the phenotype of the cells [42]. For example, MSCs proliferate on stiff hydrogels [43], whereas neuronal stem cells prefer to grow on soft hydrogels [44]. A few reports have addressed the mechanical properties of Fmoc-dipeptide hydrogels. Hence, a dynamic frequency sweep was first performed at 4 °C and 37 °C. As shown in Fig. 1B which presents G′ values at 37 °C and 4 °C, the Fmoc-FF + 30%Fmoc-RGD hydrogel stiffened upon heating. So, a dynamic temperature sweep was obtained at pH 8.5 and 7.4, reflecting the temperature-dependency of mechanical moduli (G′) (Fig. 1C). The gel stiffness could be causally related to the intermolecular interactions between the amino acids (L-phenylalanine moieties). Moreover, during the heating step, aggregated fibers were re-dissolved and locked in the self-assembled structures, raising effective fiber concentration and gel stiffness. Stiffening of the hydrogels upon temperature enhancement is in line with our observations [45, 46].

In the previous works of authors, it was reported that Fmoc-dipeptide hydrogels exhibited cell type-dependent biological activity, with higher cell proliferation in HUVEC or MDA-MB231 cells than in WJ-MSCs, indicating the potential need for incorporation of cell-adhesion ligands into the Fmoc-dipeptide hydrogels [25]. Hence, Fmoc-RGD was incorporated into the Fmoc-FF hydrogels in the present study, suggesting a significant rise in cell attachment (Fig. 2) and proliferation (Fig. 3). Cell spreading was also greatly enhanced by Fmoc-FF + 30% Fmoc-RGD compared to Fmoc-FF alone, Fmoc-FF + 15% Fmoc-RGD, and Fmoc-FF + 30% Fmoc-RGE (Fig. 2). Furthermore, Fmoc-FF + 30% Fmoc-RGD nanofibers demonstrated significantly lower cytotoxicity at various concentrations when compared to controls (Fmoc-FF, Fmoc-RGE) (Fig. 3A). Yu et al. encapsulated human mesenchymal stem cells in RGD-modified alginate microspheres and showed that RGD-modified alginate could improve cell attachment, growth, and angiogenic growth factor expression [47]. Similarly, Rong et al*.* produced PLG hydrogels through copper-free click reaction chemistry under physiological conditions. In a 3D culture model, the research showed hydrogel-conjugated RGD motifs promoted MSC adhesion and proliferation within the hydrogels [48].

Combination therapies could be more effective than monotherapies in ischemic diseases [13, 49]. SNAP is a well-known NO-donor with frequent use in the treatment of ROS-mediated diseases [50]. NO has been shown to stimulate MSC proliferation and differentiation into endothelial cells, an essential strategy for tissue regeneration [20]. In the present study, the therapeutic efficiency of the SNAP-loaded Fmoc-FF + 30%Fmoc-RGD/WJ-MSCs co-transplantation was investigated in a renal I/R injury model. To the best of the authors’ knowledge, no study has addressed the therapeutic application of co-transplantation of SNAP and WJ-MSCs in a renal I/R injury model.

The development of renal I/R injury in mouse models was confirmed by investigation of serum biochemical parameters, ROS generation, kidney tissue histopathological alterations, and gene expressions in mRNA and protein levels on the 7th day. Figure 7 demonstrates signs of tubular injury, inflammation, vacuolization, and cast in the I/R models (M). As previously demonstrated about SNAP loading in the Fmoc-FF hydrogel at a relatively low concentration (15 µM), no significant improvement was detected [22]. According to the findings of the present research, intralesional administration of free MSCs ameliorated tubular damages and inflammation in the I/R injury (Fig. 6). Despite overall efficacy, low MSC engraftment, poor delivery to the target site, and cell viability could limit the applicability of stem cell therapy. As shown in Additional file 1: Fig. S1, compared to single stem cell transplantation, co-transplantation of MSCs with Fmoc-FF + 30%Fmoc-RGD hydrogel managed to reduce cell loss and increase cell localization and proliferation. iNOS can produce toxic levels of NO. iNOS was elevated due to the renal tubular injury during hypoxia, whereas eNOS and VEGF showed a decline as protective enzymes and angiogenic growth factors [26].

Based on the findings, groups treated with WJ-MSCs showed attenuated I/R-induced renal dysfunction after seven days, whereas the SNAP-loaded Fmoc-FF + Fmoc-RGD/WJ-MSCs group exhibited the highest protection (Fig. 4, *P* < 0.0001). Although SCr level decreased in almost all treatment groups, the changes were not significant (Fig. 4B). This is consistent with previous reports expressing the SCr peak within 24 h of reperfusion, followed by a gradual decline to a baseline value [51, 52]. These reports are also in line with pathological findings, suggesting a significant decrement in inflammation, tubular injury, cast, and vacuolization (Fig. 5, *P* < 0.0001) which can be assigned to a significant inhibition in ROS production (Fig. 4C, *P* < 0.01) and iNOS expression (Figs. 6 and 7B, *P*<0.001) as confirmed by RT-PCR and IHC analyses. Compared to the I/R control, SNAP-loaded Fmoc-FF + Fmoc-RGD/WJ-MSCs expressed more eNOS (Figs. 6 and 7A, *P* < 0.001), indicating regeneration of injured endothelial tissue. Interestingly, when MSCs were combined with either SNAP or the SNAP-loaded Fmoc-FF + 30%Fmoc-RGD hydrogel, VEGF expression increased, reflecting the induction of the angiogenesis healing process (Figs. 6 and 7C, *P* < 0.0001). Peptide hydrogels could compensate for NO deficiency and accelerate the regeneration of vascular endothelial injuries, thus, increasing the eNOS expression. The released NO could also inhibit cytokine-induced iNOS expression and nuclear factor-κB (NF-κB) activation [53], reducing the I/R injury by attenuating oxidative stress. Moreover, the peptide hydrogel scaffold could buffer MSCs from the forces during injection and build proper NO-releasing support, creating a protective microenvironment for cell survival. Regarding the localized and sustained release of NO, the current study indicated that SNAP-loaded Fmoc-FF + Fmoc-RGD/WJ-MSCs can better mediate regeneration of renal I/R injuries compared to either SNAP or MSCs alone.

## Conclusion

The SNAP-loaded Fmoc-FF + Fmoc-RGD hydrogel was developed to improve the therapeutic effects of MSCs in renal I/R injury. The prepared system demonstrated prominent features such as shear-thinning property, biological compatibility, high engraftment and retention of WJ-MSCs, and consistent NO release, resulting in improved therapeutic efficiency in mouse models compared to individual treatment with free SNAP and WJ-MSCs. It was found that intralesional administration of the SNAP-loaded Fmoc-FF + Fmoc-RGD/WJ-MSCs significantly increased the survival rate of transplanted WJ-MSCs and promoted endothelium-related gene expression in WJ-MSCs. WJ-MSCs' proangiogenic ability was substantially increased in the co-transplantation system, resulting in superior neovascularization and regeneration of ischemic tissues. However, further studies are required to ascertain the roles of other nephrology endpoints and biomarkers, such as inflammatory cytokines, in renal I/R injury. Finally, the development of a strategy to enhance MSC proangiogenic activity with biomaterials such as SNAP-loaded Fmoc-FF + Fmoc-RGD could provide a practical solution to resolve the problems in the clinical applications of MSCs in I/R injuries.

## Supplementary Information

**Additional file 1** Fluorescent bioimaging of mice received normal saline (A), MSCs (B), or MSCs plus Fmoc-FF+30%Fmoc-RGD (C); arrows show the site of MSC accumulation in the renal I/R animals after intralesional injecions.

## Data Availability

All data generated or analyzed during this study are included in this published article and its Additional file 1.

## Abbreviations

AKI: Acute kidney injury
BUN: Blood urea nitrogen
CHCl_3_: Chloroform
DAB: Diaminobenzidine
DCFH-DA: 2′,7′-Dichlorofluorescein diacetate
eNOS: Endothelial nitric oxide synthase
FE-SEM: Field-emission scanning electron microscopy
FDA: Fluorescein diacetate
Fmoc-FF: Fmoc-diphenylalanine
GAPDH: Glyceraldehyde-3-phosphate dehydrogenase
H&E: Hematoxylin and eosin
hP-MSCs: Human placenta-derived mesenchymal stem cells
iNOS: Inducible nitric oxide synthase
IGF-1: Insulin-like growth factor-1
I/R: Ischemia/reperfusion
MTT: 3-(4,5-Dimethylthiazol-2-yl)-2,5-diphenyltetrazolium bromide
Nap: Naphthalene
NO: Nitric oxide
NMM: N-methyl morpholine
NF-κB: Nuclear factor-κB
PBS: Phosphate-buffered saline
PI: Propidium iodide
ROS: Reactive oxygen species
SAPs: Self-assembling peptides
SCr: Serum creatinine
SNAP: S-nitroso-n-acetyl penicillamine
SD: Standard deviation
TFA: Trifluoroacetic acid
VEGF: Vascular endothelial growth factor
WJ-MSCs: Wharton's jelly-mesenchymal stem cells
