# Supplementary material for: Integrin receptor-binding nanofibrous peptide hydrogel for combined mesenchymal stem cell therapy and nitric oxide delivery in renal ischemia/reperfusion injury
Source: Stem Cell Res Ther. 2022 Jul 26;13:344. doi: 10.1186/s13287-022-03045-1 (PMC9327234; doi:10.1186/s13287-022-03045-1)
Supplement: Supplementary file 1 — Additional file 1 Fluorescent bioimaging of mice received normal saline (A), MSCs (B), or MSCs plus Fmoc-FF+30%Fmoc-RGD (C); arrows show the site of MSC accumulation in the renal I/R animals after intralesional injecions. [file 13287_2022_3045_MOESM1_ESM.docx]

**Integrin Receptor-Binding Nanofibrous Peptide Hydrogel for Combined Mesenchymal Stem Cell Therapy and Nitric Oxide Delivery in Renal Ischemia/Reperfusion Injury**

*Haniyeh Najafi ^a^, Samira Sadat Abolmaali ^a, b^, Reza Heidari ^c^, Hadi Valizadeh ^d^, Ali Mohammad Tamaddon ^a, b, *^, Negar Azarpira ^e, *^*

**WJ-MSCs tracking in the renal ischemia/reperfusion (I/R) mice**


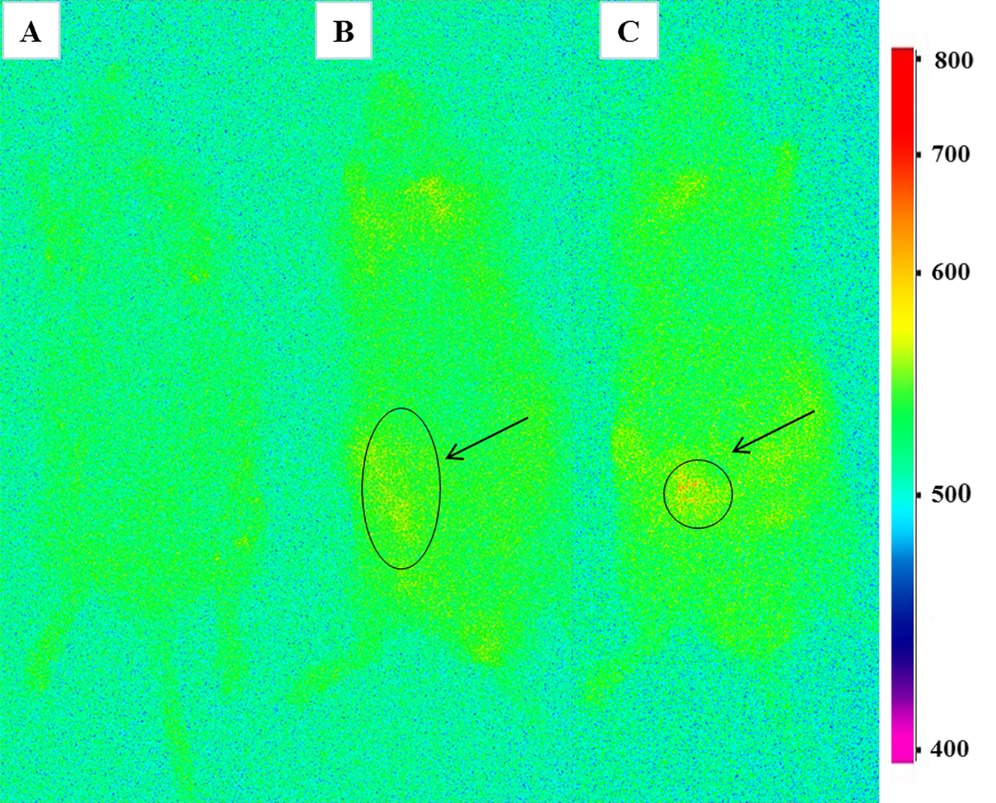


**Additional file. 1.** Fluorescent bioimaging of mice received normal saline (A), MSCs (B), or MSCs plus Fmoc-FF+30%Fmoc-RGD (C); arrows show the site of MSC accumulation in the renal I/R animals after intralesional injecions.
